# Supplementary material for: C-terminally phosphorylated p27 activates self-renewal driver genes to program cancer stem cell expansion, mammary hyperplasia and cancer
Source: Nat Commun. 2024 Jun 17;15:5152. doi: 10.1038/s41467-024-48742-y (PMC11183067; doi:10.1038/s41467-024-48742-y)
Supplement: Supplementary file 1 — Supplementary Information [file 41467_2024_48742_MOESM1_ESM.pdf]

C-terminally phosphorylated p27 activates self-renewal driver genes to program cancer stem cell expansion, mammary hyperplasia and cancer

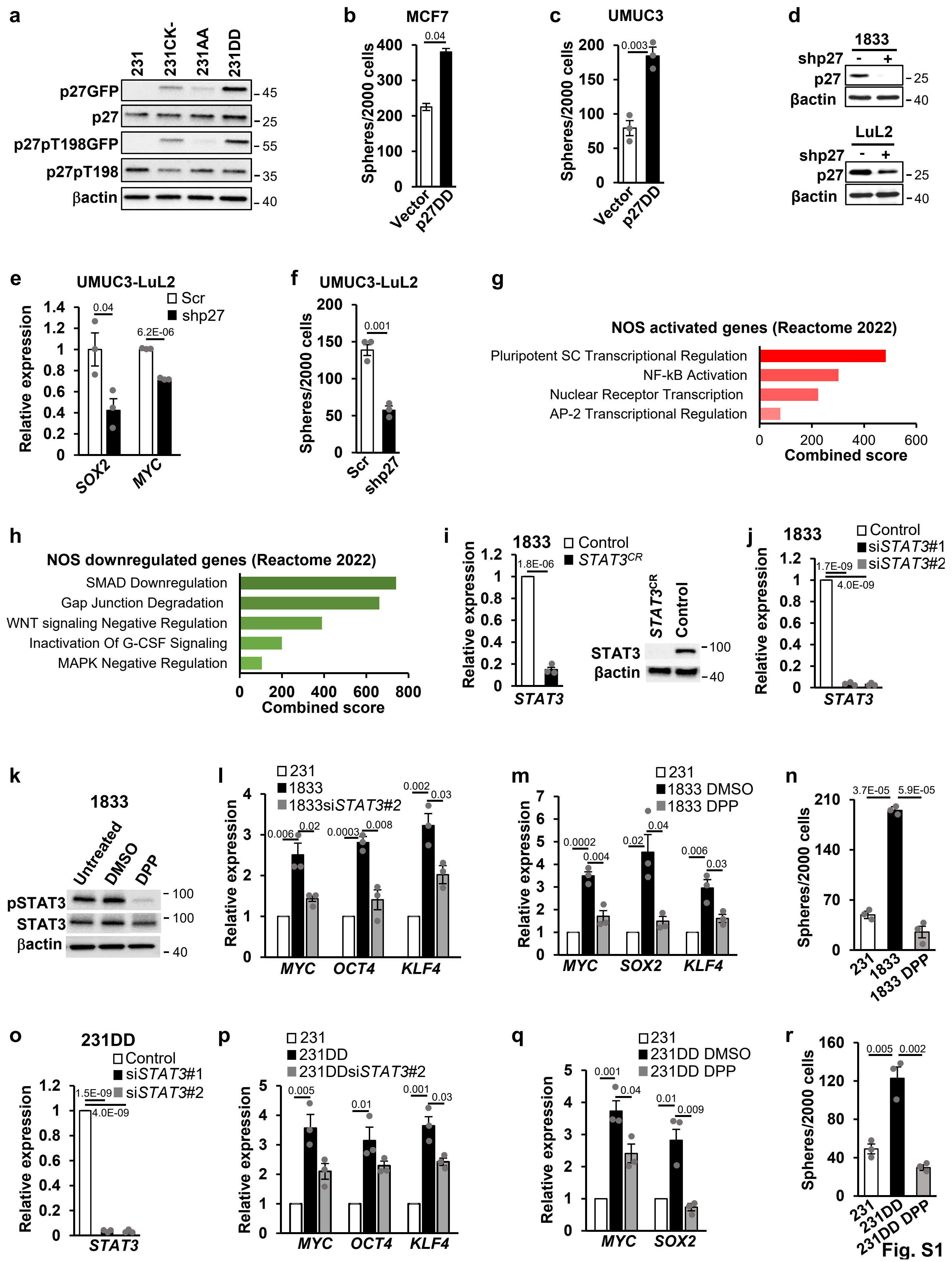

a
231
231CK-
231AA
231DD
p27GFP
p27
p27pT198GFP
p27pT198
βactin
b
MCF7
Spheres/2000 cells
Vector
p27DD
0.04
c
UMUC3
0.003
d
1833
shp27
LuL2
e
UMUC3-LuL2
Relative expression
Scr
shp27
SOX2
MYC
0.04
6.2E-06
f
0.001
g
NOS activated genes (Reactome 2022)
Pluripotent SC Transcriptional Regulation
NF-kB Activation
Nuclear Receptor Transcription
AP-2 Transcriptional Regulation
Combined score
h
NOS downregulated genes (Reactome 2022)
SMAD Downregulation
Gap Junction Degradation
WNT signaling Negative Regulation
Inactivation Of G-CSF Signaling
MAPK Negative Regulation
Combined score
i
1833
Control
STAT3CR
1.8E-06
STAT3
j
siSTAT3#1
siSTAT3#2
1.7E-09
4.0E-09
k
Untreated
DMSO
DPP
pSTAT3
l
1833siSTAT3#2
OCT4
KLF4
m
1833 DMSO
1833 DPP
n
1833 DPP
3.7E-05
5.9E-05
o
231DD
1.5E-09
p
231DDsiSTAT3#2
q
231DD DMSO
231DD DPP
r
231DD DPP
0.005
0.002

Fig. S1

Fig. S1 | p27pTpT drives stem cell properties via STAT3.

a Western blots show p27 and p27pT198 with β-actin control in 231, 231p27CK-, 231p27CK-AA, and 231p27CK-DD

b-c Effect of p27CK-DD induction on sphere formation in MCF7 (**b**) and in UMUC3-LUL2 cells (**c**).

d Western blots confirm p27 depletion in 1833 (top) and LUL2 (bottom) cells.

e-f Effect of p27 depletion on embryonic stem cell transcription factors (ES-TF) expression (**e**) and sphere formation (**f**) in UMUC3-LUL2 cells

g-h Pathway analysis of p27-regulated NOS (Nanog, Oct4, and Sox2) target genes.

i *STAT3* CRISPR knockout confirmed in 1833 cells.

j *STAT3* siRNA knockdown confirmed in 1833 cells.

k 1833 cells treated with STAT3 inhibitor (5, 15-DPP) for 48 h.

l-m Effect of *STAT3* siRNA knockdown (**l**) and STAT3 inhibitor (**m**) on p27pTpT-upregulated ES-TFs expression in 1833 cells.

n Sphere assay in 1833 cells treated with STAT3 inhibitor.

o *STAT3* siRNA knockdown confirmed in 231DD cells.

p-q Effect of *STAT3* siRNA knockdown (**p**) and STAT3 inhibitor treatment (**q**) on p27pTpT-upregulated ES-TFs expression in 231p27CK-DD cells.

r Sphere assay in 231DD cells treated with STAT3 inhibitor.

All graphs show mean ± SEM from N = 3 biological replicate assays. p values were represented by paired one-tailed Student's T Test. Source data are provided as a Source Data file.

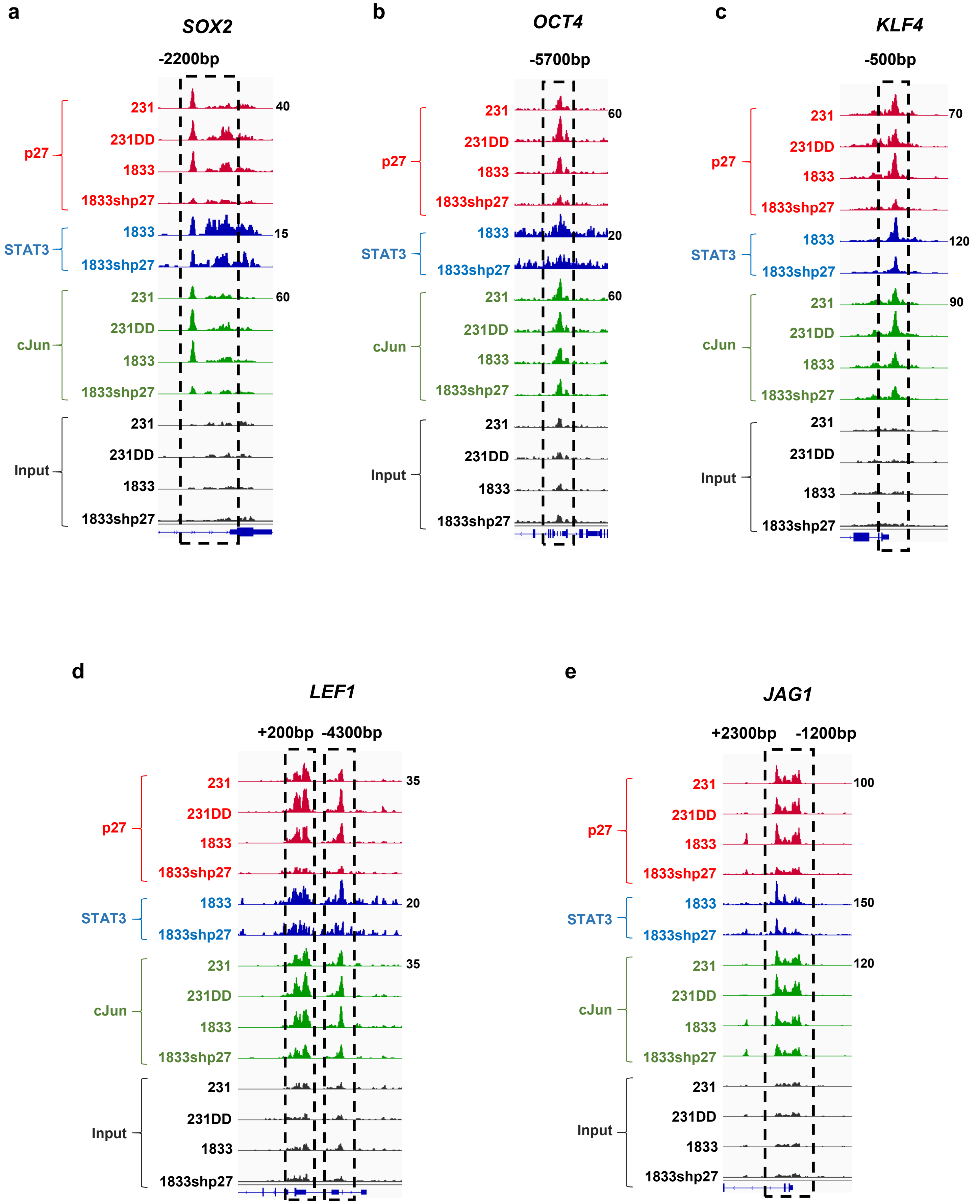
a
SOX2
-2200bp
p27
231
231DD
1833
1833shp27
40
STAT3
1833
1833shp27
15
cJun
231
231DD
1833
1833shp27
60
Input
231
231DD
1833
1833shp27
b
OCT4
-5700bp
p27
231
231DD
1833
1833shp27
60
STAT3
1833
1833shp27
20
cJun
231
231DD
1833
1833shp27
60
Input
231
231DD
1833
1833shp27
c
KLF4
-500bp
p27
231
231DD
1833
1833shp27
70
STAT3
1833
1833shp27
120
cJun
231
231DD
1833
1833shp27
90
Input
231
231DD
1833
1833shp27
d
LEF1
+200bp
-4300bp
p27
231
231DD
1833
1833shp27
35
STAT3
1833
1833shp27
20
cJun
231
231DD
1833
1833shp27
35
Input
231
231DD
1833
1833shp27
e
JAG1
+2300bp
-1200bp
p27
231
231DD
1833
1833shp27
100
STAT3
1833
1833shp27
150
cJun
231
231DD
1833
1833shp27
120
Input
231
231DD
1833
1833shp27

Fig. S2

Fig. S2 | p27-dependent STAT3, cJun enrichment at +/- 5 kb of TSS of the SC and CSC driver genes. a-e p27, STAT3, and cJun co-occupied peaks at *SOX2* (**a**), *OCT4* (**b**), *KLF4 (***c**), *LEF1* (**d**), and *JAG1* (**e**) promoters

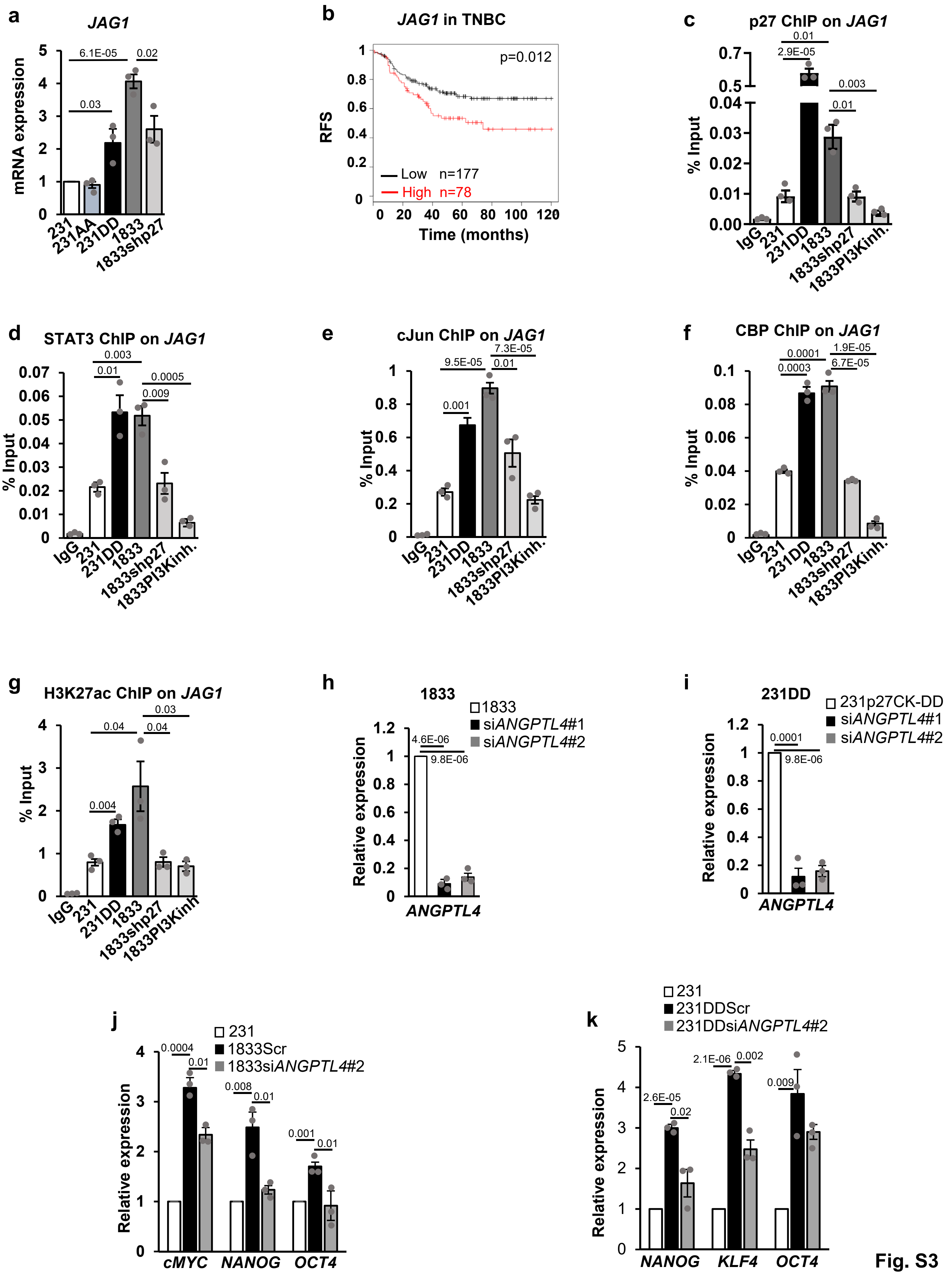
a
JAG1
mRNA expression
6.1E-05
0.02
0.03
231
231AA
231DD
1833
1833shp27
b
JAG1 in TNBC
RFS
p=0.012
Low n=177
High n=78
Time (months)
c
p27 ChIP on JAG1
% Input
0.01
2.9E-05
0.003
0.01
IgG
231
231DD
1833
1833shp27
1833PI3Kinh.
d
STAT3 ChIP on JAG1
% Input
0.003
0.01
0.0005
0.009
e
cJun ChIP on JAG1
% Input
9.5E-05
7.3E-05
0.01
0.001
f
CBP ChIP on JAG1
% Input
0.0001
1.9E-05
0.0003
6.7E-05
g
H3K27ac ChIP on JAG1
% Input
0.03
0.04
0.04
0.004
IgG
231
231DD
1833
1833shp27
1833PI3Kinh
h
1833
1833
siANGPTL4#1
siANGPTL4#2
Relative expression
4.6E-06
9.8E-06
ANGPTL4
i
231DD
231p27CK-DD
siANGPTL4#1
siANGPTL4#2
Relative expression
0.0001
9.8E-06
ANGPTL4
j
231
1833Scr
1833siANGPTL4#2
Relative expression
0.0004
0.01
0.008
0.01
0.001
0.01
cMYC
NANOG
OCT4
k
231
231DDScr
231DDsiANGPTL4#2
Relative expression
2.6E-05
0.02
2.1E-06
0.002
0.009
NANOG
KLF4
OCT4

Fig. S3

Fig. S3 | p27/STAT3/cJun co-activate *JAG1.*

a *JAG1* mRNA expression.

b KM plot shows relapse-free Triple Negative Breast Cancer (TNBC) Relapse Free Survival (RFS) with *JAG1* differential expression.

c-g ChIPqPCR shows p27 (**c**) STAT3 (**d**), cJun (**e**), and CBP (**f**) binding and H3K27ac (**g**) at +2kb of the *JAG1* TSS, at a site where p27, STAT3 and cJun each bind on ChIPseq.

h-i *ANGPTL4* siRNA knockdown confirmed by qPCR in 1833 (**h**) and 231p27CK-DD cells (**i**).

j-k Effect of *ANGPTL4* depletion on p27-driven increase in embryonic stem cell transcription factors (ES-TFs) expression in 1833 (**j**) and in 231p27CK-DD (**k**).

All graphs show mean ± SEM from N = 3 biological replicate assays. p-values were represented by paired one-tailed Student’s T Test. Source data are provided as a Source Data file.

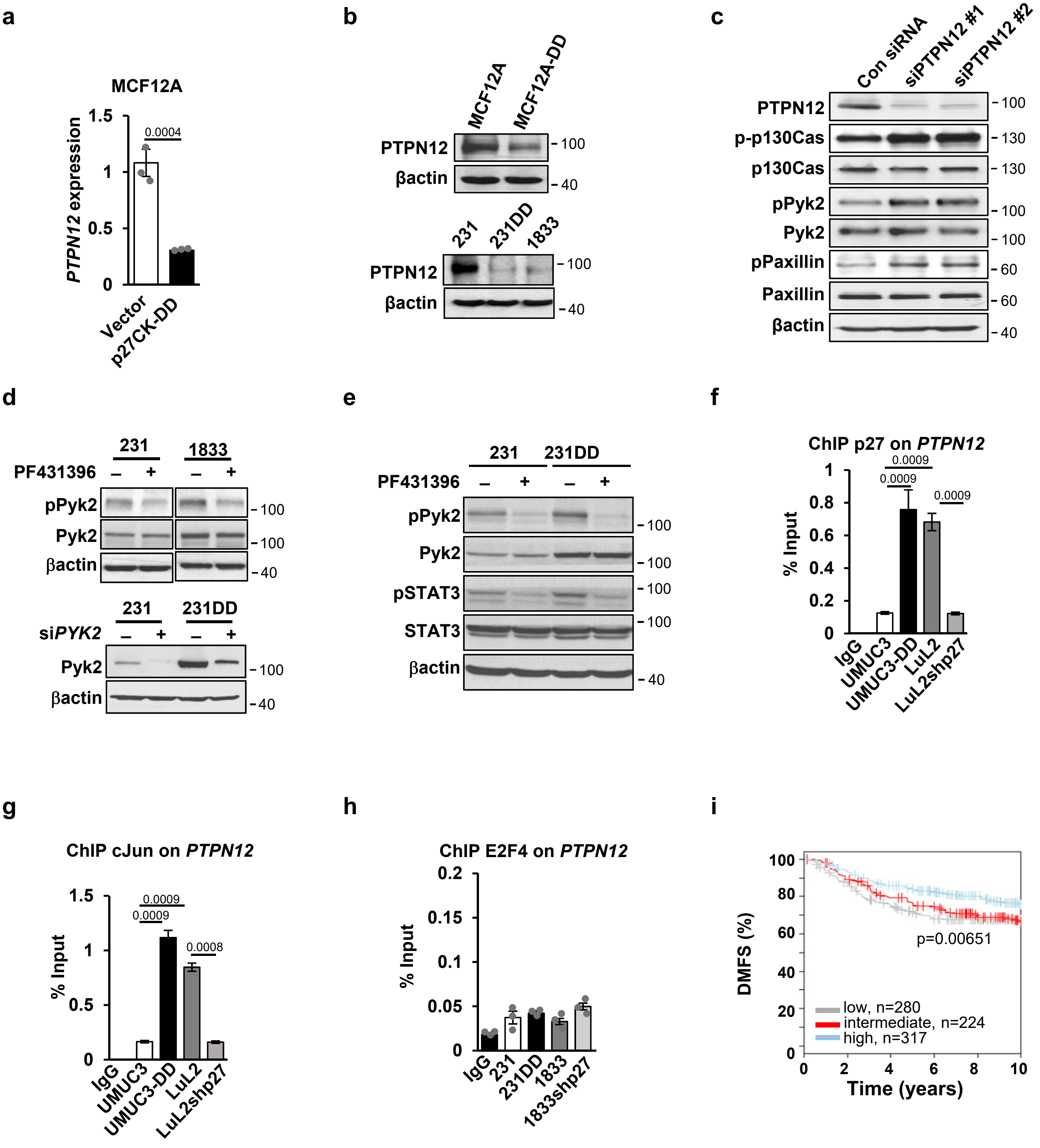
a
MCF12A
PTPN12 expression
0.0004
Vector
p27CK-DD
b
MCF12A
MCF12A-DD
PTPN12
βactin
231
231DD
1833
c
Con siRNA
siPTPN12 #1
siPTPN12 #2
PTPN12
p-p130Cas
p130Cas
pPyk2
Pyk2
pPaxillin
Paxillin
βactin
d
PF431396
siPYK2
e
pSTAT3
STAT3
f
ChIP p27 on PTPN12
% Input
0.0009
IgG
UMUC3
UMUC3-DD
LuL2
LuL2shp27
g
ChIP cJun on PTPN12
0.0008
h
ChIP E2F4 on PTPN12
1833shp27
i
DMFS (%)
p=0.00651
low, n=280
intermediate, n=224
high, n=317
Time (years)

Fig. S4

Fig. S4 | p27/cJun co-repress *PTPN12.*

a *PTPN12* expression in p27CK-DD transduced MCF12A cells.

b Western blots show PTPN12 level in MCF12A, MCF12Ap27CK-DD (top), 231, 231DD, and 1833 cells (bottom).

c Western blot depicts effect of *PTPN12* knockdown on P130Cas, Pyk2, Paxillin, and their phosphorylation level.

d Western blot confirmed effect of Pyk2 kinase inhibitor treatment on phospho-Pyk2 level (top) and confirmed *PYK2* siRNA knockdown (bottom).

e Effect of Pyk2 inhibitor treatment on phospho-STAT3 (Y705).

f-g ChIPqPCR analysis with anti-p27 (**f**) and anti-cJun antibodies (**g**) at the +2 kb site of *PTPN12* TSS in indicated cells.

h ChIPqPCR analysis for E2F4 bindings at the +2 kb site of *PTPN12* TSS in indicated cells.

All graphs show mean ± SEM from N = 3 biological replicate assays. p-values were represented by paired one-tailed Student's T Test.

i KM plot shows Distant metastasis-free survival (DMFS) with *PTPN12* differential expression.

Source data are provided as a Source Data file.

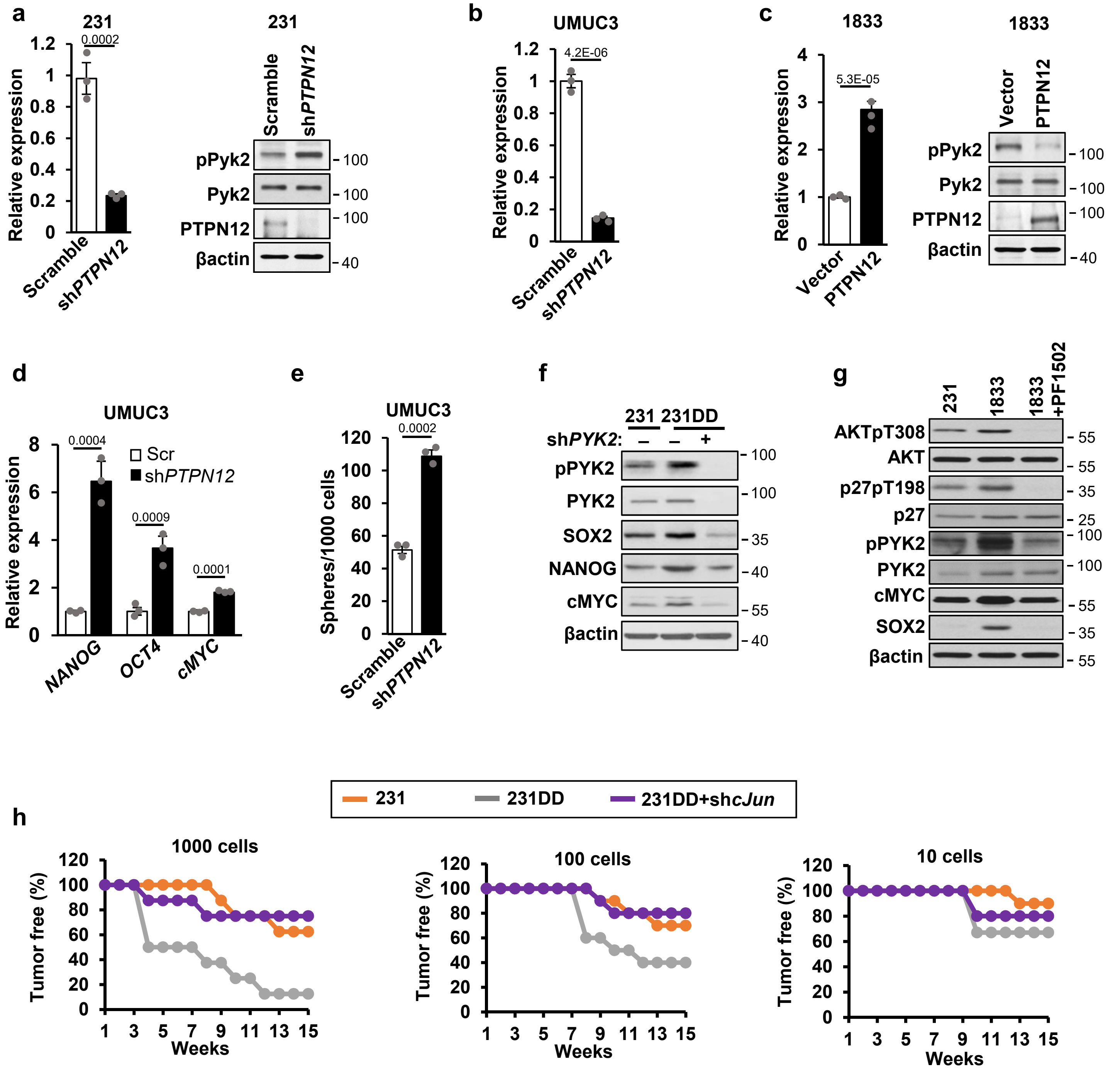

i

T-ISC frequency

Group	Limiting dilution (Tumors/Implantation)			Frequency	Pairwise Difference p value
	1000 cell	100 cell	10 cell		
**231**	**3/8**	**3/10**	**1/12**	**1/1,032**	
**231DD**	**7/8**	**6/10**	**4/12**	**1/175**	**0.0028 Vs 231** ↑
**231DDsh*cJun***	**2/8**	**2/10**	**3/12**	**1/1,122**	**<0.0001 vs 231DD** ↓

Fig. S5

Fig. S5 | p27pTpT increases T-ISC via cJun.

a-b qPCR confirmed *PTPN12* knockdown in 231 (**a**) and UMUC3 cells (**b**).

c qPCR and western blot confirmed *PTPN12* overexpression in 1833 cells.

d-e Effects of *PTPN12* knockdown on embryonic stem cell transcription factors (ES-TFs) expression (**d**) and sphere formation (**e**) in indicated cells. All graphs show mean ± SEM from N = 3 biological replicate assays. p-values were represented by paired one-tailed Student's T Test.

f Western blot shows effect of *PYK2* knockdown on p27pTpT-driven ES-TFs upregulation.

g Western blot depicts effect of PI3K inhibitor treatment on p27pTpT-driven ES-TFs upregulation.

h Effect of cJun knockdown on p27pTpT-driven increase of the Tumor Initiating Stem Cell (T-ISC) frequency. Graphs showing tumor free mice (%).

i T-ISC frequency was calculated by L-Calc Limiting Dilution Software (http://www.stemcell.com/en/Products/All-Products/LCalc-Software.aspx) from STEMCELL Technologies.

Source data are provided as a Source Data file.

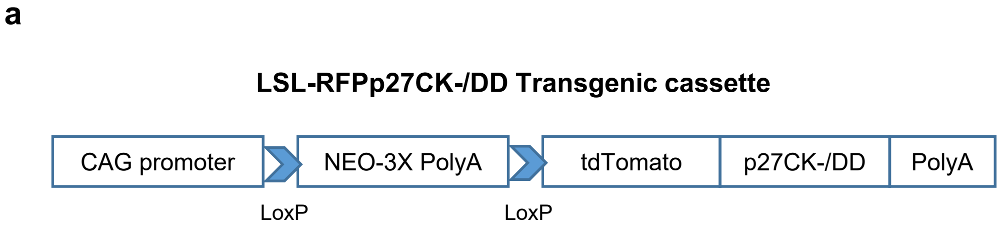

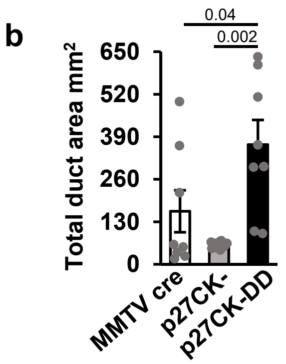

c

strain	Tumor bearing mice	% Tumors formed	p value vs cre
Cre	3/14	21.4%	-
CK-	6/14	43%	ns
DD	12/14	86%	0.001

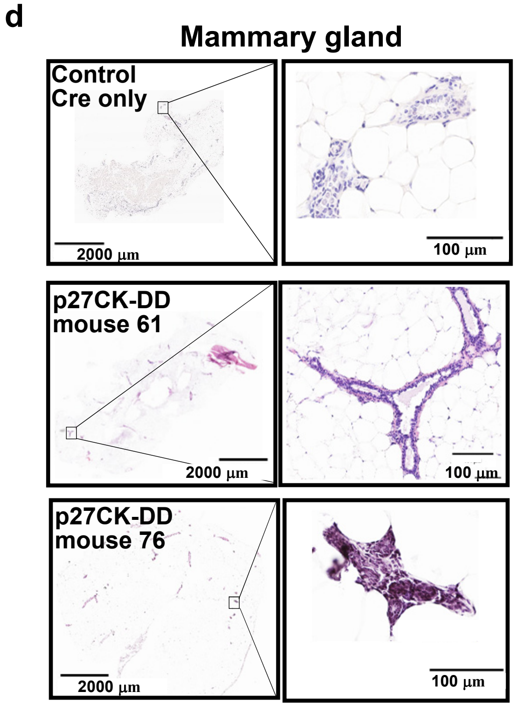

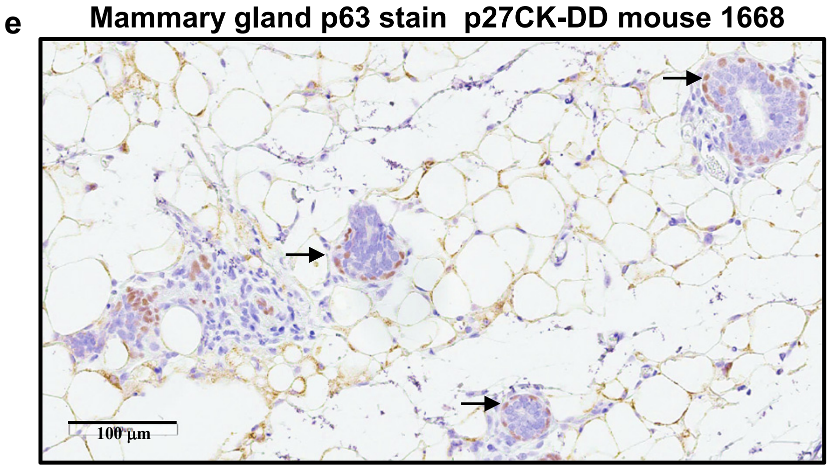

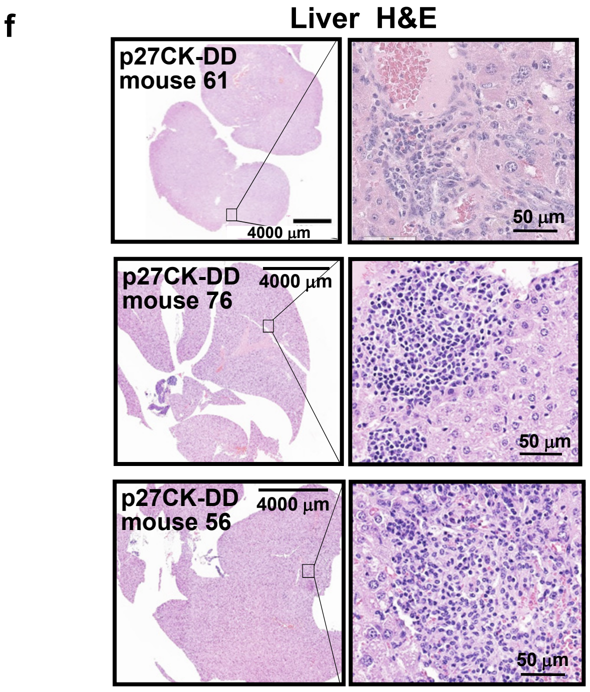

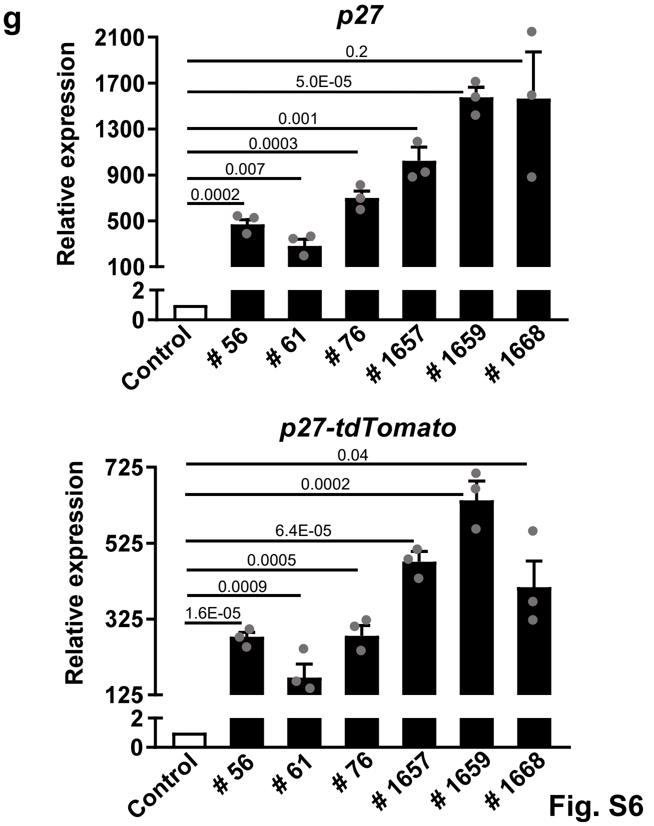

Fig. S6

Fig S6 | p27CK-DD increases mammary duct area, microinvasive cancers that metastasize to liver.

a Schematics of transgenic p27CK- and p27CK-DD expression targeting vectors.

b Duct area was quantitated from whole mounts and graphed as mean %+/-SEM. p-values were represented by one-way ANOVA with post hoc 2 by 2 comparisons using Tukey correction. N = 8 mice for MMTVCre controls, N = 8 mice for TGp27CK-, N = 8 mice for TGp27CK-DD

c Table shows tumor formation in MMTV cre, MMTV Cre X p27CK-, and MMTV Cre X p27CK-DD mice.

d Mammary gland hematoxylin and eosin (H&E) staining from MMTV Cre control mouse and the indicated p27CK-DD MG.

e p63 staining of breast myoepithelial cells is seen at the perimeter of normal ducts (upper right arrow) and is interrupted in the tumor area in the lower left. This is an expanded view of the mammary tumor from p27CK-DD mouse 1668 shown in Fig 7d.

f H&E staining of the indicated MMTV Cre X p27CK-DD mice livers showing representative micro metastases.

g qPCR shows transgenic *p27CK-DD* expression using TGp27-specific primers and primers spanning the tdTomato and p27 sequences in metastatic liver compared to control liver. N = 5 control mice and N=6 TGp27CK-DD mice. All graphs show mean ± SEM. Each TGp27CK-DD mice is compared by the average of 5 control mice. p-values were represented by paired one-tailed Student's T Test.

Source data are provided as a Source Data file.

Table S1. Primer sequence

Gene	Forward	Reverse
c-MYC (qPCR primer)	GTGGAAAAGAGGCAGGCTCC	CTTGACCCTCTTGGCAGCAG
SOX2 (qPCR primer)	ACCAGCTCGCAGACCTACAT	ACCAGCTCGCAGACCTACAT
OCT4 (qPCR primer)	TCAGCCAAACGATCTGC	TTCGCTTTCTCTTTCGGGCC
NANOG (qPCR primer)	CAGACCTGGTGCACCCAATC	CTTCCAAGGCAGCCTCCAAG
KLF4 (qPCR primer)	CCCACACTTGTGATTACGC	GGTAAGGTTTCTCACCTGTG
STAT3 (qPCR primer)	CAGTGACAGCTTCCCAATGG	ATGTGATTCTTTGCTGGCCG
ANGPTL4 (qPCR primer)	AGAAGCAGCACCTGCGAAT	CTCTTTCTTCGGGCAGGCTT
JAG1 (qPCR primer)	ACCTTCAACCTCAAGGCCAG	CGCCTCCACAAGCAACGTAT
PTPN12 (qPCR primer)	TGAAGATTCACCTCCTCCC	CCGCTGGATGATCACATTTC
p27 (qPCR primer)	TAGCGGAGCAATGCGCAGGA	AACCGGCATTTGGGGAACCGT
p27 (PCR primer)	ACTACTACGTGGACACCAAGCTG	GGGGTTTGTGATTCTGAGCATC
GAPDH	ATCAAGTGGGGCGATGCTG	ACCCATGACGAACATGGGG
c-MYC (ChIPqPCR primer)	AAAGAACGGAGGGAGGGATC	GCGAGTTAGATAAAGCCCCG
JAG1 (ChIPqPCR primer)	GTGGACCCTGAGCCGAAG	TCAAAGTGTGCCTCAAGGAG
ANGPTL4 (ChIPqPCR primer)	GAGAATGAGCTGGCTTGGAG	AGCCTGCCAGGAAGAACTC
PTPN12 (ChIPqPCR primer)	TGGAGAAGTGGCCTTAGGGT	ATTGAGCCACTGCACTCCAA
