# Supplementary material for: C-terminally phosphorylated p27 activates self-renewal driver genes to program cancer stem cell expansion, mammary hyperplasia and cancer
Source: Nat Commun. 2024 Jun 17;15:5152. doi: 10.1038/s41467-024-48742-y (PMC11183067; doi:10.1038/s41467-024-48742-y)
Supplement: Supplementary file 2 — Reporting Summary [file 41467_2024_48742_MOESM2_ESM.pdf]

nature portfolio

Corresponding author(s): Joyce Slingerland

Last updated by author(s): Apr 22, 2024

Reporting Summary

Nature Portfolio wishes to improve the reproducibility of the work that we publish. This form provides structure for consistency and transparency in reporting. For further information on Nature Portfolio policies, see our Editorial Policies and the Editorial Policy Checklist.

Statistics

For all statistical analyses, confirm that the following items are present in the figure legend, table legend, main text, or Methods section.

n/a	Confirmed	
☐	☒	The exact sample size (*n*) for each experimental group/condition, given as a discrete number and unit of measurement
☐	☒	A statement on whether measurements were taken from distinct samples or whether the same sample was measured repeatedly
☐	☒	The statistical test(s) used AND whether they are one- or two-sided<br>*Only common tests should be described solely by name; describe more complex techniques in the Methods section.*
☐	☒	A description of all covariates tested
☐	☒	A description of any assumptions or corrections, such as tests of normality and adjustment for multiple comparisons
☐	☒	A full description of the statistical parameters including central tendency (e.g. means) or other basic estimates (e.g. regression coefficient) AND variation (e.g. standard deviation) or associated estimates of uncertainty (e.g. confidence intervals)
☐	☒	For null hypothesis testing, the test statistic (e.g. *F*, *t*, *r*) with confidence intervals, effect sizes, degrees of freedom and *P* value noted<br>*Give P values as exact values whenever suitable.*
☒	☐	For Bayesian analysis, information on the choice of priors and Markov chain Monte Carlo settings
☒	☐	For hierarchical and complex designs, identification of the appropriate level for tests and full reporting of outcomes
☒	☐	Estimates of effect sizes (e.g. Cohen's *d*, Pearson's *r*), indicating how they were calculated

Our web collection on statistics for biologists contains articles on many of the points above.

Software and code

Policy information about availability of computer code

Data collection	Novaseq6000
Data analysis	Analysis softwares and packages:<br>ENCODE3 ChIPSeq pipeline https://www.encodeproject.org/chip-seq/transcription_factor/<br>FastQC (version 0.11.8) https://www.bioinformatics.babraham.ac.uk/projects/fastqc/<br>Trim Galore (version 0.4.4) https://www.bioinformatics.babraham.ac.uk/projects/trim_galore/<br>bowtie (version 2.3.3.1) https://bowtie-bio.sourceforge.net/bowtie2/index.shtml<br>PicardTools (version 2.1.1) https://broadinstitute.github.io/picard/<br>peakqualtools (version 1.2.2) https://github.com/kundajelab/phantompeakqualtools<br>MACS2 (version 2.2.1) https://github.com/macs3-project/MACS/releases<br>bedtools (version 2.26.0) https://bedtools.readthedocs.io/en/latest/<br>ChipPeakAnno (version 3.17) https://bioconductor.org/packages/release/bioc/html/ChIPpeakAnno.html<br>deepTools python package (version 2.5.3) https://deeptools.readthedocs.io/en/develop/index.html<br>ImageJ https://imagej.net/ij/download.html<br>QU path (version v0.4.3) https://qupath.github.io<br>GraphPad Prism (version 9.5.1) https://www.graphpad.com/

For manuscripts utilizing custom algorithms or software that are central to the research but not yet described in published literature, software must be made available to editors and reviewers. We strongly encourage code deposition in a community repository (e.g. GitHub). See the Nature Portfolio guidelines for submitting code & software for further information.

Data

Policy information about availability of data

All manuscripts must include a data availability statement. This statement should provide the following information, where applicable:

- Accession codes, unique identifiers, or web links for publicly available datasets
- A description of any restrictions on data availability
- For clinical datasets or third party data, please ensure that the statement adheres to our policy

The RNA-seq and ChIP-seq data reported in this paper have been deposited at GEO and are publicly available. All remaining data is available in the Article, Supplementary and Source data files.
STAT3 ChIP-seq data generated for this study are deposited in GEO under GSE233351 accession number and are publicly available.To review GEO accession GSE233351: Go to https://www.ncbi.nlm.nih.gov/geo/query/acc.cgi?acc=GSE233351
The RNAseq and the p27 and cJun ChIp-seq data are deposited in GEO under GSE112446 accession number and are publicly available. To review GEO accession GSE112446: Go to https://www.ncbi.nlm.nih.gov/geo/query/acc.cgi?acc=GSE112446
Source data are provided with this paper in the Source Data file.

Research involving human participants, their data, or biological material

Policy information about studies with human participants or human data. See also policy information about sex, gender (identity/presentation), and sexual orientation and race, ethnicity and racism.

Reporting on sex and gender	N/A
Reporting on race, ethnicity, or other socially relevant groupings	N/A
Population characteristics	N/A
Recruitment	N/A
Ethics oversight	N/A

Note that full information on the approval of the study protocol must also be provided in the manuscript.

Field-specific reporting

Please select the one below that is the best fit for your research. If you are not sure, read the appropriate sections before making your selection.

[x] Life sciences   [ ] Behavioural & social sciences   [ ] Ecological, evolutionary & environmental sciences

For a reference copy of the document with all sections, see nature.com/documents/nr-reporting-summary-flat.pdf

Life sciences study design

All studies must disclose on these points even when the disclosure is negative.

Sample size	The sample sizes were based on our previous experiences with similar analyses. We used 8 -12 mice per experimental group for all studies. The sample size are indicated in the figure legends and methods section.
Data exclusions	No data were excluded from the analysis.
Replication	The individual mice used in each experiment , n=8-12 comprise individual biologic repeats for each experimental group tested. All attempts at replication were successful.
Randomization	N/A Animal were treated in uniform condition and no drug were used. Thus no need for randomization.
Blinding	N/A Animal were treated in uniform condition and no drug were used. Thus no need for blinding.

Reporting for specific materials, systems and methods

We require information from authors about some types of materials, experimental systems and methods used in many studies. Here, indicate whether each material, system or method listed is relevant to your study. If you are not sure if a list item applies to your research, read the appropriate section before selecting a response.

Materials & experimental systems

n/a	Involved in the study	
	☒	Antibodies
	☒	Eukaryotic cell lines
☒		Palaeontology and archaeology
	☒	Animals and other organisms
☒		Clinical data
☒		Dual use research of concern
☒		Plants

Methods

n/a	Involved in the study	
	☒	ChIP-seq
	☒	Flow cytometry
☒		MRI-based neuroimaging

Antibodies

Antibodies used

Mouse monoclonal anti-p27Kip1 BD Transduction Cat# 610241
Rabbit monoclonal anti-p27Kip1 (clone D69C12) Cell Signaling Cat# 3686s
Rabbit polyclonal anti-phospho-p27Kip1 (T198) R&D systems Cat# AF3994
Mouse monoclonal anti-STAT3 (clone 124H6) Cell Signaling Cat# 9139s
Rabbit monoclonal anti-STAT3 (clone D3Z2G) Cell Signaling Cat# 12640s
Rabbit monoclonal anti-phospho-STAT3 (Tyr705) (clone D3Z2G) Cell Signaling Cat# 9145s
Mouse monoclonal anti-Pyk2 (clone 5E2) Cell Signaling Cat# 3480s
Rabbit monoclonal anti-phospho-Pyk2 (Tyr402) Cell Signaling Cat# 3291s
Rabbit monoclonal anti-CBP (clone D6C5) Cell Signaling Cat# 7389s
Rabbit monoclonal anti-p130 (clone E1L9H) Cell Signaling Cat# 13846s
Rabbit polyclonal anti-phospho-p130 (Tyr410) Cell Signaling Cat# 4011s
Rabbit monoclonal anti-c-Myc (clone D84C12) Cell Signaling Cat# 5605s
Rabbit monoclonal anti-Sox2 (clone D6D9) Cell Signaling Cat# 3579s
Rabbit monoclonal anti-Nanog (clone D2A3) Cell Signaling Cat# 8822s
Rabbit monoclonal anti-cJun (clone 60A8) Cell Signaling Cat# 9165s
Rabbit monoclonal anti-phospho-cJun (Ser63) (clone 54B3) Cell Signaling Cat# 2361s
Rabbit polyclonal anti-PTPN12 Abcam Cat# ab289859
Rabbit polyclonal anti-SIN3A Abcam Cat# ab3479
Rabbit polyclonal anti-YY1 Abcam Cat# ab12132
Rabbit polyclonal anti-HDAC1 (clone H-51) Santa Cruz Cat# sc-7872
Rabbit monoclonal Acetyl-Histone H3 (Lys27) (clone D5E4) Cell Signaling Cat# 8173s
Mouse monoclonal anti-Actin (clone AC-15) Sigma-Aldrich Cat# A1978
Normal rabbit IgG Cell Signaling Cat# 2729s
Normal mouse IgG Santa Cruz Cat# sc-2025
Anti-rabbit IgG (H+L) HRP conjugate Promega Cat# W4011
Anti-mouse IgG (H+L) HRP conjugate Promega Cat# W4021
Mouse anti-CD44-APC (clone G44-26) BD Biosciences Cat# 559942
Mouse anti-CD24-PE BD Biosciences Cat# 560991
Rabbit polyclonal anti- phospho-p27Kip1 (T198) ThermoFisher Cat# 36862
Goat Anti-Rabbit IgG Antibody (H+L), Biotinylated Vector Laboratories Cat# BA-1000-1.5
Mouse monoclonal anti human Cytokeratin (clone AE1/AE3) Dako Cat# M3515
Rabbit polyclonal Purified anti-p63 (ΔN) Antibody (clone Poly6190) Biolegend Cat#619002

Validation

All antibodies used in this study are well-recognized clones and commercially available. Antibodies have been validated both by the manufacturer and citations provided for the scientific literature. Validation data for each antibody can be accessed on the respective manufacturer's homepage.

Eukaryotic cell lines

Policy information about cell lines and Sex and Gender in Research

Cell line source(s)

MDA-MB-231, Luciferase conjugate J. Massague (Minn AJ. et al., 2005)
MDA-MB-231-p27CK-DD, Luciferase conjugate J.M. Slingerland (Zhao D. et al., 2015)
MDA-MB-231-1833, Luciferase conjugate J. Massague (Minn AJ. et al., 2005)
MDA-MB-231-1833-shp27 Luciferase conjugate J.M. Slingerland (Yoon H. et al, 2019)
MDA-MB-231-1833+STAT3 CRISPR This paper
MDA-MB-231+shPTPN12, Luciferase conjugate This paper
MDA-MB-231-1833-PTPN12, Luciferase conjugate This paper
UMUC3, Luciferase conjugate (Nitz MD. et al., 2008)
UMUC3-p27CK-DD, Luciferase conjugate J.M. Slingerland (Zhao D. et al., 2015)
UMUC3-LuL2, Luciferase conjugate (Nitz MD. et al., 2008)
UMUC3-LuL2-shp27 J.M. Slingerland (Zhao D. et al., 2015)
UMUC3+shPTPN12, Luciferase conjugate This paper

MCF-12A ATCC Cat#CRL-10782
MCF-12A-p27CK-DD J.M. Slingerland (Zhao D. et al., 2015)
MCF7 Marc Lippman Lab

Authentication	The authenticity of the cell lines was confirmed through short-tandem repeat (STR) fingerprinting analysis by Georgetown University Tissue Culture Shared Resource (TCSR).
Mycoplasma contamination	Cells were tested for mycoplasma monthly and before in vivo experiments. All cell lines tested negative for mycoplasma contamination.
Commonly misidentified lines (See ICLAC register)	No commonly misidentified cell lines were used in the study.

Animals and other research organisms

Policy information about studies involving animals; ARRIVE guidelines recommended for reporting animal research, and Sex and Gender in Research

Laboratory animals

For transgenic animal:
pCAG-LSL-RFPp27CK- and pCAG-LSL-RFPp27CK-transgenes were microinjected into the C57B6 mice zygote pronuclear in the Animal Models Shared Resource of University of Miami. Founder CAG-LSL-RFPp27CK- transgenic mice and founder CAG-LSL-RFPp27CK-DD transgenic mice were selected and their genotypes were verified by PCR amplification of a 350bp DNA fragment spanning the RFP and p27 sequences. To activate transgenic expression of p27CK- and p27CK-DD, the pCAG-LSL-p27CK-(KpnI-) and pCAG-LSL-p27CK-DD(KpnI-) transgenic mice were bred with MMTV-CreA mice (purchased from Jackson lab). The genotypes of bigenic MMTV-Cre; p27CK- or MMTVCre;p27CK-DD progeny were verified by PCR analysis of genomic DNA from tail biopsies using Cre-specific and either RPF or p27 primers. Virgin female mice were recovered at 18 months of age. A total of 42mice were used for the transgenic studies. All mice were housed and bred in accordance with institutional guidelines on a 12h light/dark cycle with constant ambient temperature (22–24 °C) and humidity (46-48%). Animal protocols were reviewed and approved by the Institutional Animal Care and Use Committee (IACUC) of the University of Miami (protocol 17-166) or and of Georgetown University (protocol 2021-0014). All murine experiments complied with our IACUC maximum tumor size of 1000mm3 and the maximal tumor size was not exceeded.

For In vivo limiting dilution T-ISC assay:
All animal research was conducted in accordance with the University of Miami Animal Care Committee. For limiting dilution T-ISC assays, 5 week-old female Balb/c nude mice were purchased from Charles River Laboratories (Boston, MA, USA). Limiting dilutions of 10, 100, and 1000 cells were each suspended in 10 mg/ml Matrigel with Hanks' Balanced Salt Solution (HBSS; Lonza, 10-547F), and injected into the 4rth inguinal mammary fat pad (n=12, 10, 8 mice per group). A total of 240 mice were used for the TISC experiment. All mice were housed and bred in accordance with institutional guidelines on a 12h light/dark cycle with constant ambient temperature (22–24 °C) and humidity (46-48%). Mice were euthanized per IACUC guidelines. Tumor size was measured twice/week, and tumor volumes were estimated as length × width × width × 0.5. T-ISC frequency was calculated by L-Calc Limiting Dilution Software (STEMCELL™). Animal protocols were reviewed and approved by the Institutional Animal Care and Use Committee (IACUC) of the University of Miami (protocol 17-166) or and of Georgetown University (protocol 2021-0014). All murine experiments complied with our IACUC maximum tumor size of 1000mm3 and the maximal tumor size was not exceeded.

Wild animals	No wild animals were used in this study.
Reporting on sex	Only female mice were used in this study
Field-collected samples	This study did not involve field-sample collections.
Ethics oversight	Animal protocols were reviewed and approved by the Institutional Animal Care and Use Committee (IACUC) of the University of Miami or of Georgetown University.

Note that full information on the approval of the study protocol must also be provided in the manuscript.

Plants

Seed stocks	*Report on the source of all seed stocks or other plant material used. If applicable, state the seed stock centre and catalogue number. If plant specimens were collected from the field, describe the collection location, date and sampling procedures.*
Novel plant genotypes	*Describe the methods by which all novel plant genotypes were produced. This includes those generated by transgenic approaches, gene editing, chemical/radiation-based mutagenesis and hybridization. For transgenic lines, describe the transformation method, the number of independent lines analyzed and the generation upon which experiments were performed. For gene-edited lines, describe the editor used, the endogenous sequence targeted for editing, the targeting guide RNA sequence (if applicable) and how the editor was applied.*
Authentication	*Describe any authentication procedures for each seed stock used or novel genotype generated. Describe any experiments used to assess the effect of a mutation and, where applicable, how potential secondary effects (e.g. second site T-DNA insertions, mosiacism, off-target gene editing) were examined.*

ChIP-seq

Data deposition

☒ Confirm that both raw and final processed data have been deposited in a public database such as GEO.

☒ Confirm that you have deposited or provided access to graph files (e.g. BED files) for the called peaks.

Data access links<br>*May remain private before publication.*	STAT3 ChIP-seq data are deposited in GEO under GSE233351 accession number and are publicly available as of the date of publication. To review GEO accession GSE233351:<br>Go to https://www.ncbi.nlm.nih.gov/geo/query/acc.cgi?acc=GSE233351<br>Enter token mpcxueikzhubjyh into the box<br>The RNAseq and the p27 and cJun ChIp-seq data are deposited in GEO under GSE112446 accession number and are publicly available. To review GEO accession GSE112446: Go to https://www.ncbi.nlm.nih.gov/geo/query/acc.cgi?acc=GSE112446
Files in database submission	Please see above
Genome browser session<br>(e.g. UCSC)	N/A

Methodology

Replicates	One biological replicate
Sequencing depth	ChIP libraries were sequenced to ~40million reads.
Antibodies	Rabbit monoclonal anti-STAT3 (clone D3Z2G) Cell Signaling Cat# 12640s<br>Rabbit monoclonal anti-p27Kip1 (clone D69C12) Cell Signaling Cat# 3686s<br>Rabbit monoclonal anti-cJun (clone 60A8) Cell Signaling Cat# 9165s
Peak calling parameters	The ChIP-Seq analysis was setup following ENCODE3 ChIPseq pipeline (https://www.encodeproject.org/chip-seq/transcription_factor/). Initial quality was inspected by FastQC (version 0.11.8) (https://www.bioinformatics.babraham.ac.uk/projects/fastqc/), and adapters were trimmed with Trim Galore (version 0.4.4) (https://www.bioinformatics.babraham.ac.uk/projects/trim_galore/) leaving preprocessed reads for global alignment. Genome alignment was performed by bowtie (version 2.3.3.1) (https://bowtie-bio.sourceforge.net/bowtie2/index.shtml) against the hg19 version of the human genome. After alignment, unaligned reads were removed from the alignment file, and reads that were multimapped were corrected to keep the best alignment. Duplicate reads were marked and removed from the aligned bam file using PicardTools (version 2.1.1) (https://broadinstitute.github.io/picard/). To start assessing quality of alignment, the PCR bottleneck coefficient was calculated based off this final aligned bam file. Further QC was calculated including the cross correlation coefficient with phantom peakqualtools (version 1.2.2) (https://github.com/kundajelab/phantompeakqualtools) 104,105. After QC, peaks were initially called with MACS2 (version 2.2.1) (https://github.com/macs3-project/MACS/releases) 106 with general default parameters.<br>Peaks that belonged to the blacklist region were then excluded from further analysis, and peaks were filtered with q-value threshold of e-10. Overlapping peaks were then calculated using customized scripts based off bedtools (version 2.26.0) (https://bedtools.readthedocs.io/en/latest/) 107 to calculate the reads that belonged to individual samples, and overlapping peaks. Resulting peaks were annotated to the hg19 Ensembl reference using the R package ChipPeakAnno (version 3.17) (https://bioconductor.org/packages/release/bioc/html/ChIPpeakAnno.html) 108,109. Peaks that were +/- 5Kb away from gene transcription start site (TSS) were assigned the gene. Intensity plots were calculated using the deepTools python package (version 2.5.3) (https://deeptools.readthedocs.io/en/develop/index.html) 110 to calculate the coverage matrix and plot the resulting signal intensities. Gene start sites for these plots were set to +/- 2Kb of the start site of the peak for the coverage matrix. Peaks were then split out into different categories with 0.75 thresholds of intensity values from the comparison sample. Those with >0.75 were said to be gained, those <0.25 were said to be lost peaks in the comparison with the in between intensities were classified as retained peaks.
Data quality	Initial quality was inspected by FastQC (version 0.11.8) (https://www.bioinformatics.babraham.ac.uk/projects/fastqc/). To start assessing quality of alignment, the PCR bottleneck coefficient was calculated based off this final aligned bam file. Further QC was calculated including the cross correlation coefficient with phantom peakqualtools (version 1.2.2) (https://github.com/kundajelab/phantompeakqualtools). Peaks that belonged to the blacklist region were excluded from further analysis, and peaks were filtered with q-value threshold of e-10 and fold-change > 6. The difference of signal intensity in terms of all the selected genes between different cell lines (1833 vs.231 and 231-DD vs. 231) was evaluated with paired t-test and p values were subsequently adjusted with BH method.
Software	ENCODE3 ChIPSeq pipeline https://www.encodeproject.org/chip-seq/transcription_factor/<br>FastQC (version 0.11.8) https://www.bioinformatics.babraham.ac.uk/projects/fastqc/<br>Trim Galore (version 0.4.4) https://www.bioinformatics.babraham.ac.uk/projects/trim_galore/<br>bowtie (version 2.3.3.1) https://bowtie-bio.sourceforge.net/bowtie2/index.shtml<br>PicardTools (version 2.1.1) https://broadinstitute.github.io/picard/<br>peakqualtools (version 1.2.2) https://github.com/kundajelab/phantompeakqualtools<br>MACS2 (version 2.2.1) https://github.com/macs3-project/MACS/releases<br>bedtools (version 2.26.0) https://bedtools.readthedocs.io/en/latest/<br>ChipPeakAnno (version 3.17) https://bioconductor.org/packages/release/bioc/html/ChIPpeakAnno.html<br>deepTools python package (version 2.5.3) https://deeptools.readthedocs.io/en/develop/index.html

ImageJ https://imagej.net/ij/download.html
QU path (version v0.4.3) https://qupath.github.io
GraphPad Prism (version 9.5.1) https://www.graphpad.com/
All original code has been deposited at Zenodo and is publicly available as of the date of publication. DOI:10.5281/zenodo.7966839
To access the record please open the following private link:
https://zenodo.org/record/7966839?token=eyJhbGciOiJIUzUxMiIsImV4cCI6MTY4ODA3NTk5OSwiaWF0IjoxNjg1NDY4MTgyfQ.eyJkYXRhIjp7InJlY2lkIjo3OTY2ODM5fSwiaWQiOjM0MDMyLCJybmQiOiJkOGM1ZTM0ZSJ9.Z3CrZ8lkTTvx7-GUBBavYfJleEzvx1IepH8UfRbdgJ7_uMdSp67u_ce0IIFwKoy9mzQbr24-IR5XWpwct2AktA

Flow Cytometry

Plots

Confirm that:

- [x] The axis labels state the marker and fluorochrome used (e.g. CD4-FITC).
- [x] The axis scales are clearly visible. Include numbers along axes only for bottom left plot of group (a 'group' is an analysis of identical markers).
- [ ] All plots are contour plots with outliers or pseudocolor plots.
- [x] A numerical value for number of cells or percentage (with statistics) is provided.

Methodology

Sample preparation	Cells were incubated with anti-CD44-APC (BD Biosciences, 559942) and anti-CD24-PE (BD Biosciences, 560991) antibodies for 60 min at 4°C. After washing twice with PBS containing 0.1% bovine serum albumin (BSA), the cells were analyzed using a BD FACSCanto II Flow Cytometer (BD Biosciences).
Instrument	BD FACSCanto II Flow Cytometer (BD Biosciences)
Software	FlowJo software (TreeStar)
Cell population abundance	At least 20,000 cells were collected for analysis and data are representative of 3 or more biologic repeats.
Gating strategy	Gating and setup of flow was carried out per protocols established for Aldefluor and for CD44 and CD24 surface marker assessment per published protocols. References are provided in the methods section.

- [x] Tick this box to confirm that a figure exemplifying the gating strategy is provided in the Supplementary Information.
